# Evaluation of Short-Term Exposure to 2.4 GHz Radiofrequency Radiation Emitted from Wi-Fi Routers on the Antimicrobial Susceptibility of *Pseudomonas aeruginosa* and *Staphylococcus aureus*

**DOI:** 10.31661/gmj.v9i0.1580

**Published:** 2020-03-14

**Authors:** Samad Amani, Mohammad Taheri, Mohammad Mehdi Movahedi, Mohammad Mohebi, Fatemeh Nouri, Alireza Mehdizadeh

**Affiliations:** ^1^Shiraz University of Medical Sciences, Shiraz, Iran; ^2^Department of Medical Microbiology, Faculty of Medicine, Hamadan University of Medical Sciences, Hamadan, Iran; ^3^Department of Medical Physics and Medical Engineering, School of Medicine, Shiraz University of Medical Sciences, Shiraz, Iran; ^4^Ionizing and Non-ionizing Radiation Protection Research Center (INIRPRC), Shiraz University of Medical Sciences, Shiraz, Iran; ^5^School of Medicine, Shiraz University of Medical Sciences, Shiraz, Iran; ^6^Department of Pharmaceutical Biotechnology, School of Pharmacy, Hamadan University of Medical Sciences, Hamadan, Iran

**Keywords:** Pseudomonas aeruginosa, Staphylococcus aureus, Radiofrequency, Drug Resistance

## Abstract

**Background:**

Overuse of antibiotics is a cause of bacterial resistance. It is known that electromagnetic waves emitted from electrical devices can cause changes in biological systems. This study aimed at evaluating the effects of short-term exposure to electromagnetic fields emitted from common Wi-Fi routers on changes in antibiotic sensitivity to opportunistic pathogenic bacteria.

**Materials and Methods:**

Standard strains of bacteria were prepared in this study. Antibiotic susceptibility test, based on the Kirby-Bauer disk diffusion method, was carried out in Mueller-Hinton agar plates. Two different antibiotic susceptibility tests for *Staphylococcus aureus* and *Pseudomonas aeruginosa* were conducted after exposure to 2.4-GHz radiofrequency radiation. The control group was not exposed to radiation.

**Results:**

Our findings revealed that by increasing the duration of exposure to electromagnetic waves at a frequency of 2.4 GHz, bacterial resistance increased against *S. aureus* and *P. aeruginosa*, especially after 24 hours (P<0.05).

**Conclusion:**

The use of electromagnetic waves with a frequency of 2.4 GHz can be a suitable method for infection control and treatment.

## Introduction

During the last decades, due to the increase in the telecommunication systems uses, such as wireless fidelity (Wi-Fi) and mobile communication (GSM), several changes have occurred in the natural environment. Wi-Fi radiation, as a part of non-ionizing radiation in the electromagnetic spectrum, usually produce radiofrequency waves with a frequency of 2.4 GHz [1]. It is evident that electromagnetic radiation either ionizing or non-ionizing can affect biological systems via thermal or non-thermal effects [2]. As a result, the electromagnetic effects on the biological functions of living microorganisms represent an interesting area of research regarding the environmental impacts on human health. It has been reported that electromagnetic field (EMFs) can either negatively [3-6] or positively [5,7,8] affect cell growth and viability, as well as bacterial sensitivity to antibiotics, depending on its physical parameters, such as frequency, exposure time, intensity and magnetic flux density and bacterial strain. The non-thermal effects of Wi-Fi exposure on different strains of bacteria have been studied [5–11]. Overall, the effects of EMFs on the biological systems have been investigated in several studies [1,9]. However, the results have been controversial, and different cell responses have been reported. In this regard, our previous study reported that Wi-Fi exposure could not only change the antibacterial susceptibility but also cause changes in the growth rate of pathogenic bacteria such as *Listeria monocytogenes* and *Escherichia coli* [10]. Another study revealed that Wi-Fi radiation significantly enhanced the susceptibility of *Klebsiella pneumoniae* to various antibiotics [11]. Accordingly, it is necessary to evaluate the EMF effects on the bacteria and also find strategies to control antibacterial susceptibility in the clinical laboratories or even in the environment [12,13]. *Pseudomonas aeruginosa* is a Gram-negative, oxidase-positive, non-stimulating, aerobic bacterium. It accounts for 11% to 23% of hospital infections, especially in patients with cystic fibrosis, burn or immune deficiency, and dependence on respiratory equipment [14]. The development of multi-drug resistant (MDR) strains of *P. aeruginosa* is critical in the acquisition and development of resistance in Gram-negative bacteria [15,16]. On the other hand, *Staphylococcus aureus* is a Gram-positive bacterium and also one of the most common pathogens in human communities and hospitals. Despite the availability of effective antibiotics, this pathogen considered the most common cause of skin and surgical wound infections [17]. The present study aimed to evaluate the effects of short-term exposure of Wi-Fi emitted from routers, on the susceptibility of *S. aureus* and *P. aeruginosa*, as opportunistic microorganisms, to various antibiotics.

## Materials and Methods

###  Antimicrobial Agents

In this study, the following antibiotics were evaluated against *P. aeruginosa*; levofloxacin (5 μg), piperacillin (100 μg), aztreonam (30 μg), ciprofloxacin (5 μg), imipenem (10 μg), amikacin (30 μg), cefotaxime (30 μg), and ceftriaxone (30 μg). Also, ampicillin (10 μg), vancomycin (30 μg), cotrimoxazole (30 μg), tetracycline (30 μg), amikacin (30 μg), levofloxacin (5 μg), piperacillin (100 μg), and ceftriaxone (30 μg) were used for *S. aureus*. The antibiotic susceptibility pattern was performed according to the Clinical and Laboratory Standards Institute guidelines. The Kirby-Bauer disc diffusion method was applied in Mueller-Hinton Agar (MHA; Biolife, Italy). The antibiotic disks were purchased by ROSCO Diagnostica (DK-2630 Taastrup, Denmark).

###  Antimicrobial Susceptibility Testing

The two bacterial strains, including *P. aeruginosa* ATCC 27853, and *S. aureus* ATCC 25923 were obtained. Fresh bacterial cultures (0.5 McFarland turbidity, 1.5×108 CFU) were prepared separately. Each bacterial suspension was poured on the plate. Afterward, different types of antibiotic disks were placed on the plates for each bacterium and kept in incubators at 37°C. The test group was exposed to a Wi-Fi router at 2.4 GHz in a 14.5 cm distance (far field) [18], while the control group had no exposure to such radiation. In the test group, Wi-Fi exposure was achieved at intervals of 2, 4, 6, 8, 10, and 24 hours. The antibiotic susceptibility results were measured and recorded before and after exposure to waves emitted from the Wi-Fi router. The inhibition zone diameters were also recorded (mm).

###  Statistical Analysis

 The inhibition zone diameters in the exposed and non-exposed groups were analyzed using IBM SPSS Inc. version 22. The tests were carried out in triplicate, and the non-parametric Mann-Whitney U test was performed for data analysis. P-value<0.05 was considered as significant differences.

## Results

###  Effects of Radiation Emitted from Wi-Fi Routers on P. aeruginosa

**Figure 1:**
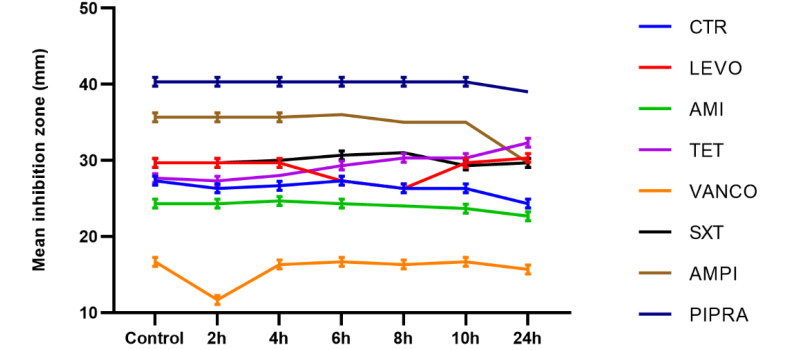
The mean inhibition zone diameter of *P. aeruginosa *bacteria before and after exposure to Wi-Fi radiation.

**Figure 2:**
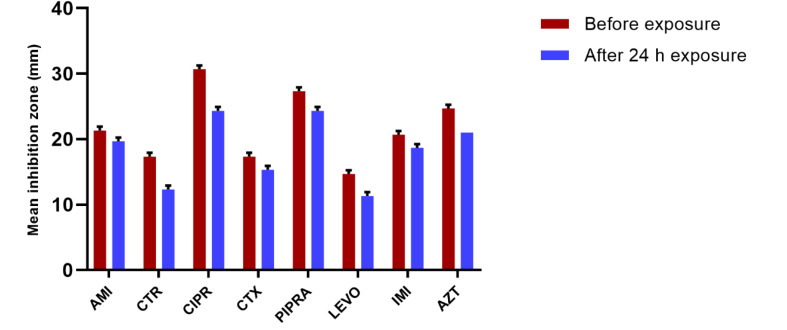
The mean inhibition zone diameter of *P. aeruginosa *after 24 hours of exposure to Wi-Fi radiation.

In *P. aeruginosa* bacteria, the inhibition zone diameters decreased significantly compared to with the control group at the following times: after two hours of Wi-Fi exposure emitted from the Wi-Fi router for ceftriaxone; after four hours of treatment with ceftriaxone and cefotaxime; after six hours of treatment with ceftriaxone, cefotaxime, and piperacillin; after eight and ten hours of treatment with ceftriaxone, cefotaxime, piperacillin, ciprofloxacin, aztreonam; and finally after 24 hours of exposure with all antibiotics. All of the observed changes were statistically significant (P<0.05). The mean inhibition zone diameters of *P. aeruginosa* are presented in Figure-1. According to this Figure, bacteria, which were exposed to radiofrequency radiation, showed remarkable changes in susceptibility to antibiotics in comparison with the control group at different exposure intervals. As shown in Figure-1, reduction in the inhibition zone diameter was related to increased resistance to antibiotics; therefore, after 24 hours of exposure to 2.4-GHz radiation, resistance patterns were determined for all antibiotics; changes in antibacterial susceptibility to ciprofloxacin were evident (Figure-2).

###  Effects of Wi-Fi Router Radiation on S. aureus

**Figure 3:**
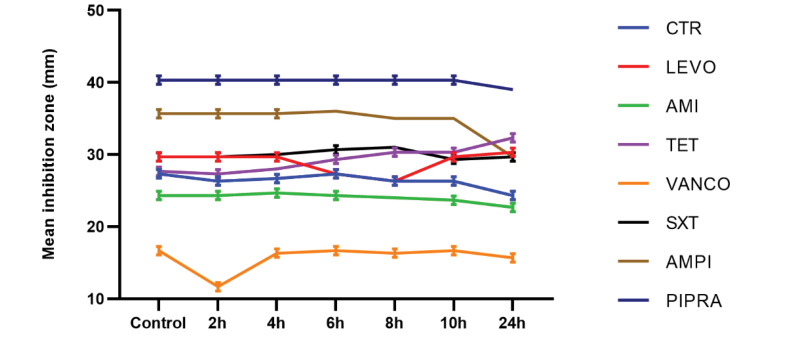
The mean inhibition zone diameter of S. aureus bacteria before and after exposure to Wi-Fi radiation.

**Figure 4:**
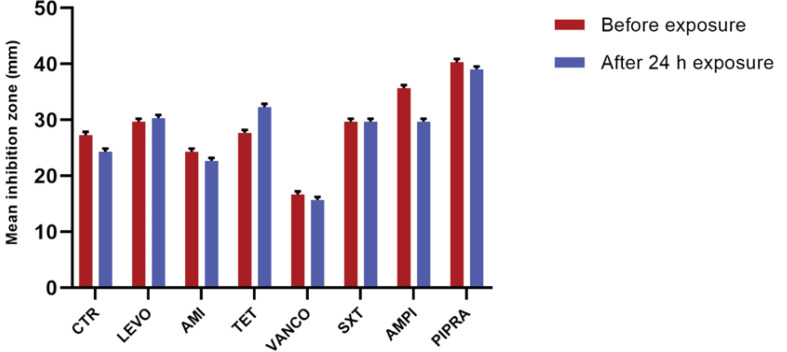
The mean inhibition zone diameter of *S. aureus *after 24 hours of exposure to Wi-Fi radiation.

Although the inhibition zone diameter of *S. aureus* decreased after two hours of exposure to Wi-Fi router radiation at a frequency of 2.4 GH, this change was not statistically significant for vancomycin. Our findings showed that after four hours of exposure to radiation, no significant difference was observed in susceptibility to the studied antibiotics in comparison with the control group. Also, after six hours of exposure to levofloxacin, the mean inhibition zone diameter decreased, whereas it increased for tetracycline in comparison with the control group. These changes were significant for tetracycline and levofloxacin after four hours of exposure (P<0.05). Based on the findings, after eight hours of exposure, the mean inhibition zone of levofloxacin and tetracycline decreased and increased, respectively; however, it remained unchanged for cotrimoxazole, compared with the control group. According to Figure-3, after 10 hours of exposure to radiation, the mean inhibition zone diameter of tetracycline was significantly different from the non-exposed group (P<0.05). After 24 hours of Wi-Fi exposure, the inhibition zone diameters for ceftriaxone, amikacin, ampicillin, and piperacillin were decreased (Figure-4). On the other hand, it increased significantly for tetracycline in comparison with the control group (P<0.05). The results of antimicrobial susceptibility tests for *S. aureus* are presented in Figure-3. *S. aureus *bacteria, which were exposed to radiofrequency radiation for different periods, showed remarkable changes in susceptibility, compared with antibiotics used in the control group. Consequently, *S. aureus* bacteria, which were exposed to radiofrequency radiation at 2.4 GHz, showed resistance to antibiotics, including ceftriaxone, amikacin, vancomycin, ampicillin, and piperacillin, compared with the control group; these changes were more significant for tetracycline and vancomycin.

## Discussion

The aim of the present study was to examine the short-term exposure effect to radiation emitted from a Wi-Fi router on the sensitivity of pathogenic microorganisms to various antibiotics. The results of this study showed that there are significant changes in the resistance of *S. aureus* and *P. aeruginosa *to different antibiotics due to exposure to 2.4 GHz radiation after 24 hours. *P. aeruginosa* was resistant to all antibiotics after 24 hours of exposure to radiofrequency waves. Moreover, *S. aureus* was resistant to some antibiotics, such as ampicillin, amikacin, ceftriaxone, vancomycin, and piperacillin. These results are in line with the findings reported by Stansell *et al*. [19]. They showed that static magnetic fields with moderate intensity can reduce the susceptibility of *E. coli* to piperacillin after 45 minutes of exposure and make it more resistant to antibiotics. Although the duration and frequency of exposure and type of microorganism and antibiotic are different in these studies, the non-ionizing radiation source was similar; accordingly, susceptibility changes were remarkable. In the present study, by increasing the exposure time to 24 hours for 2.4-GHz waves emitted from the Wi-Fi router, the sensitivity of *P. aeruginosa* to different antibiotics decreased in comparison with the control group. In our previous study [11], the effect of exposure to 2.4-GHz electromagnetic waves emitted from Wi-Fi routers on *K. pneumoniae* was evaluated for 4.5 hours. The results showed the increased sensitivity of this bacterium to ceftriaxone, cefotaxime, piperacillin, imipenem, and aztreonam. The discrepancy between the results of antibacterial susceptibility tests may be attributed to the nature of bacteria (Gram-positive or Gram-negative) and duration of exposure. In contrast with our findings, Segatore *et al*. [20], in a study on the effects of low-frequency (2 mT, 50 Hz) EMFs on the antibacterial susceptibility of *E. coli *and *P. aeruginosa, *reported no remarkable differences in the antibiotic sensitivity of these bacteria in the exposed groups in comparison with the control group. Also, inconsistent findings have been reported for *S. aureus*. In the present study, after exposure to Wi-Fi radiation, these bacteria were more susceptible to certain antibiotics, such as tetracycline and levofloxacin, compared with the control group, although they were more resistant to ceftriaxone and amikacin. The mentioned finding is in line with the results of our previous study [10] on *E. coli* and *Listeria monocytogenes,* which showed the increased resistance of *E. coli *to all antibiotics after six hours of exposure to 900-MHz waves from a radiofrequency simulator; However, *L. monocytogenes* remained resistant to ciprofloxacin and cefotaxime after nine hours of exposure to 2.4-GHz waves from a Wi-Fi router; this finding is similar to the present study. On the other hand, Salmen [21]ÿÿÿÿ- reported that high EMFs, at frequencies of 900 and 1800 MHz, had no remarkable effects on *S. aureus *bacteria, treated with 30 µg of amoxicillin in the exposed group. Moreover, Belyaev [7] showed that, under particular conditions, extremely-low frequency (ELF)-EMF can be considered a non-toxic tool stimulating the growth of E. coli GE499. On the other hand, Aslanimehr *et al*. [6] evaluated the effect of ELF-EMF on the growth rate and viability of *S. aureus* and *E. coli. *A reduction was observed in the growth rate of bacteria; therefore, it was suggested that ELF-EMF may be a therapeutic approach for controlling acute and chronic infections. Two factors, which can affect antimicrobial susceptibility, are the structure and function of antibiotics. Antibiotics show antibacterial functions through different mechanisms, such as inhibition of DNA and cell-wall synthesis and prevention of dihydrofolate production and protein synthesis. In this regard, Taheri *et al*. showed that electromagnetic waves can cause some changes in the structure of efflux pumps and ion channels in the cell wall and alter their permeability to molecules entering the bacterial cell; therefore, antibacterial susceptibility of bacteria to different antibiotics changes [22]. Overall, the present study showed that 2.4-GHz waves emitted from Wi-Fi routers can change the antibacterial susceptibility of microorganisms. It seems that these waves can change the function of ion channels and efflux pumps of bacterial cell walls upon the entry of molecules into the cell; however, there are still many other unknown mechanisms that can influence bacterial responses. Several researchers have examined different effects of EMF on bacteria; these effects (e.g., bactericidal effects) vary depending on the frequency and intensity of EMF, coherence, and exposure time [23-25]. Furthermore, the phase of bacterial growth, medium ingredients, genetic properties, growth in the presence or absence of oxygen, and membrane features in bacteria should be considered [25]. It is assumed that EMF, relative to its frequency, can change antibacterial susceptibility. In this regard, a previous study reported an increase in the antibacterial effects with 51.8 GHz radiation [26]. Moreover, disruption of cation transport reduced adenosine triphosphate content [27], and different proteome contents in the membrane can enhance antibiotic susceptibility [28].

###  Study Limitations

 This research study was carried out only on the two kinds of pathogenic bacteria, and only at the frequency of the 2.4 GHz radiofrequency radiation. To extrapolate the results of the current study, further studies with different microorganisms and different exposure conditions are suggested.

## Conclusion

 It is believed that EMF, relative to its frequency, can change antibacterial susceptibility. In this regard, it is suggested that future studies investigate the effects of electromagnetic waves emitted from different devices, examine the use of different antimicrobial agents, and evaluate the effect of longer exposure time. Also, the use of electromagnetic waves with a frequency of 2.4 GHz can be considered as a suitable method for infection control and treatment.

## Acknowledgment

 This study was supported by Shiraz University of Medical Sciences, Iran. This research was a part of Mohammad Mohebi thesis (thesis No. 94.1013; ethical approval No.IR.SUMS.REC.1395.245).

## Conflict of Interest

 None declared.
